# Improving the appropriateness of antipsychotic prescribing in nursing homes: a mixed-methods process evaluation of an academic detailing intervention

**DOI:** 10.1186/s13012-017-0602-z

**Published:** 2017-05-26

**Authors:** L. Desveaux, M. Saragosa, J. Rogers, L. Bevan, H. Loshak, A. Moser, S. Feldman, L. Regier, L. Jeffs, N. M. Ivers

**Affiliations:** 1 Women’s College Research Institute, Women’s College Hospital, 76 Grenville Ave Toronto, Toronto, Ontario M5S 1B2 Canada; 2 Keenan Research Centre of the Li Ka Shing Knowledge Institute, St. Michael’s Hospital, 209 Victoria St, Toronto, Ontario Canada; 3 Centre for Effective Practice, 203 College St, Toronto, Ontario Canada; 4 Baycrest Health Sciences, 3560 Baycrest St, Toronto, Ontario Canada; 5 Family Practice Health Centre, Women’s College Research Institute, Women’s College Hospital, 76 Grenville Ave Toronto, Toronto, Ontario Canada

**Keywords:** Academic detailing, Antipsychotic medication, Inappropriate prescribing, Nursing home, Mixed methods, Consolidated Framework for Implementation Research

## Abstract

**Background:**

In 2014, nursing home administration and government officials were facing increasing public and media scrutiny around the variation of antipsychotic medication (APM) prescribing across Ontario nursing homes. In response, policy makers partnered to test an academic detailing (AD) intervention to address appropriate prescribing of APM in nursing homes in a cluster-randomized trial. This mixed-methods study aimed to explore how and why the AD intervention may have resulted in changes in the nursing home context. The objectives were to understand how the intervention was implemented, explore contextual factors associated with implementation, and examine impact of the intervention on prescribing.

**Methods:**

Administrative data for the primary outcome of the full randomized trial will not be available for a minimum of 1 year. Therefore, this paper reports the findings of a planned, quantitative interim trial analysis assessed mean APM dose and prescribing prevalence at baseline and 3 and 6 months across 40 nursing homes (18 intervention, 22 control). Patient-level administrative data regarding prescribing were analyzed using generalized linear mixed effects regression. Semi-structured interviews were conducted with nursing home staff from the intervention group to explore opinions and experiences of the AD intervention. Interviews were analyzed using the framework method, with constructs from the Consolidated Framework for Implementation Research (CFIR) applied as pre-defined deductive codes. Open coding was applied when emerging themes did not align with CFIR constructs. Qualitative and quantitative findings were triangulated to examine points of divergence to understand how the intervention may work and to identify areas for future opportunities and areas for improvement.

**Results:**

No significant differences were observed in prescribing outcomes. A total of 22 interviews were conducted, including four academic detailers and 18 nursing home staff. Constructs within the CFIR domains of Outer Setting, Inner Setting, and Characteristics of Individuals presented barriers to antipsychotic prescribing. *Intervention Source*, *Evidence Strength and Quality*, and *Adaptability* explained participant engagement in the AD intervention; nursing homes that exhibited a *Tension for Change* and *Leadership Engagement* reported positive changes in processes and communication.

**Conclusions:**

Participants described their experiences with the intervention against the backdrop of a range of factors that influence APM prescribing in nursing homes that exist at the system, facility, provider, and resident levels. In this context, the perceived credibility and flexibility of the intervention were critical features that explained engagement with and potential impact of the intervention. Development of a common language across the team to enable communication was reported as a proximal outcome that may eventually have an effect on APM prescribing rates. Process evaluations may be useful during early stages of evaluation to understand how the intervention is working and how it might work better. Qualitative results suggest the lack of early changes observed in prescribing may reflect the number of upstream factors that need to change for APM rates to decrease.

**Trial registration:**

ClinicalTrials.gov, NCT02604056

**Electronic supplementary material:**

The online version of this article (doi:10.1186/s13012-017-0602-z) contains supplementary material, which is available to authorized users.

## Background

Older adults in nursing homes experience multimorbidity and functional decline [1] which often results in a higher prevalence of polypharmacy compared to their community-dwelling contemporaries [2, 3]. An inability to detect errors in their medication and receiving care from multiple point-of-care staff across multiple settings coupled with age-related changes in pharmacokinetic and pharmacodynamic responses increase the likelihood of drug-related adverse events [4, 5]. Given their frailty and reduced physiologic reserves, any adverse event has the potential to irreversibly contribute to patient decline and premature death [6].

Inappropriate drug prescribing refers to suboptimal over-prescribing practices that introduce a greater risk of drug-related adverse events, particularly in circumstances when a safer, equally effective alternative is available [7]. Conversely, under-prescribing can also lead to adverse effects, including ongoing distress and avoidable medical events (e.g. repeat myocardial infarctions in the absence of beta blockers). Among nursing home residents with severe dementia, antipsychotic medication (APM) use was associated with suboptimal prescribing [8], with reported uses including the treatment of behavioural and psychiatric symptoms (e.g. verbal and physical aggression), emotional states (e.g. anger and sadness), and cognitive diagnoses [9]. APM use may also cause a variety of adverse events, some of which may be treated by other drugs resulting in a prescribing cascade and polypharmacy [10]. A range of negative consequences are associated with polypharmacy, including reduced functional capacity and an increased risk of adverse drug interactions, hospital admissions, and morbidity [11–13].

Multi-component educational strategies including interactive approaches such as academic detailing (AD), which utilize interactive and tailored approaches with direct feedback, appear to be the most effective at improving prescribing appropriateness [6, 14, 15]. A targeted educational intervention delivered to physicians, nurses, and other nursing home staff promoting non-pharmacological techniques and gradual APM withdrawal reduced days of APM use by 72% [16]. Notably, no associated increases in behavioural problems were observed among baseline APM users who had prescriptions discontinued for three or more months [16]. Preliminary evidence suggests that prescriber characteristics affect APM prescribing independent of resident factors and facility characteristics, underscoring the need for a flexible approach which is individualized to the intervention recipient [17].

With an increasing focus on rates of APM in nursing homes in Ontario, policy makers partnered with an academic detailing service to trial an AD intervention to address appropriate prescribing in nursing homes [18]. Specifically, the intervention aimed to reduce inappropriate prescribing, defined as “an unfavorable ratio of the risk for adverse drug events relative to potential benefits” ([14] (p.629)) and is often measured by the presence of a given medication (e.g. APM) in the absence of an evidence-based indication [e.g. severe, acute behavioural and psychological symptoms of dementia (BPSD)]. AD is a form of educational outreach in which a trained health care professional visits healthcare providers (typically physicians) in their practice environment to provide evidence-based information on a selected topic [19]. Coupled with its demonstrated influence on prescribing behaviours [20], the flexibility of AD is well-suited to these behaviours in complex environments such as nursing homes, given that the critical part of the intervention is determining and addressing individual participant needs. This study aimed to explore how and why the AD intervention worked or did not work in the nursing home context. Specifically, the objectives were to understand how the intervention was implemented, explore the contextual factors associated with implementation, and examine impact of the intervention on prescribing.

## Methods

### Study design

**Fig. 1:**
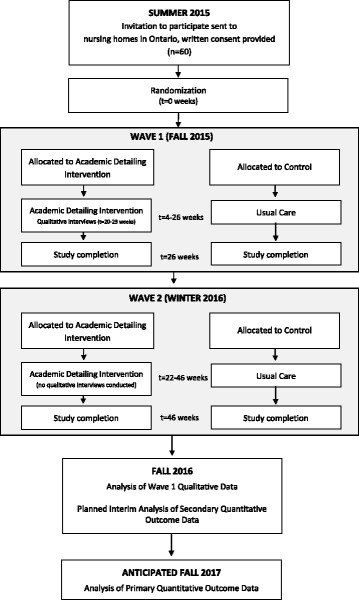
Overall study design

This mixed-methods process evaluation was embedded in a two-arm, pragmatic, cluster randomized controlled trial of an AD intervention to improve the appropriateness of APM prescribing in nursing homes, described previously [18]. Participating nursing homes were allocated using a 2:1 ratio to the full, active intervention (featuring AD offered to each prescriber and team members in the home) or standard quality improvement supports. Blinded analysis of primary outcomes will determine effectiveness, which will use population-level administrative databases to analyze prescribing outcomes at 6 months. The analyses utilize administrative data that will not be available until a minimum of 1 year following the trial, and will therefore be reported in a subsequent paper. Thus, this manuscript reports the results of a planned interim analysis of secondary outcomes alongside a qualitative process evaluation that was conducted following the completion of the intervention. A flow diagram outlining the timing of the evaluations reported in this manuscript can be found in Fig. 1. This project involved a partnership between the Ontario Ministry of Health and Long-Term Care (MOHLTC) and Ontario Medical Association (OMA) to explore opportunities to improve prescribing in Ontario [18]; therefore, the timing of the qualitative process evaluation and interim outcome analysis was planned to meet the needs of policy stakeholders to inform future funding decisions. The MOHLTC and the OMA dictated the parameters of the project and provided oversight, while the Centre for Effective Practice (CEP, https://effectivepractice.org) was responsible for the design and implementation of the AD intervention. The members of the research team (LD, MS, LJ, and NI) were responsible for conducting an external and independent process evaluation using qualitative methods.

The protocol received ethics approval from the University of Toronto and Women’s College Hospital Research Ethics Boards. The trial is registered on ClinicalTrials.gov (NLM Identifier: NCT02604056).

### Intervention

**Fig. 2:**
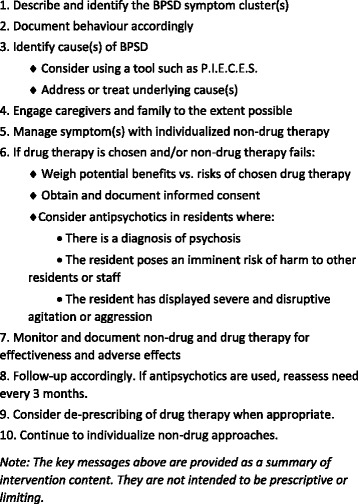
Academic detailing intervention key messages

The AD intervention included educational content to address two behaviours in nursing homes—the management of residents with BPSD and the prescribing of APM. Detailers were trained to follow a service-oriented approach, conducting individual needs assessments and providing evidence-informed information around a series of key messages targeting behaviour change (refer to Fig. 2). Key messages were developed following a literature search of clinical and implementation evidence, as well as an environmental scan to identify programs, stakeholders, and materials related to appropriate APM prescribing and managing BPSD. From these searches, the CEP extracted the key messages, building out the features, benefits, barriers, and enablers for each, prior to review and revision by a clinical working group. An eight-page discussion guide provided a synthesis of available evidence and is publicly available through the CEP website (https://effectivepractice.org/resources/academic-detailing-service/). The discussion guide was distributed directly to intervention homes by the detailers, but was also publicly available to all nursing homes in Ontario. The primary audience was defined as individuals with the most direct role in the prescribing of antipsychotic medications for residents and/or the implementation of home-wide interventions. This included physicians, medical directors, directors of care, pharmacists, nurse practitioners, nurse managers, and administrative/executive directors. The secondary audience was defined as individuals with a direct role in the care of residents with BPSD and an indirect role in the prescribing of antipsychotic medications for these residents through documenting behaviours, including nurses, social workers, personal support workers, and other d providers. Engaging the secondary audience was dependent upon the expressed interest of the home, the individual providers in question, and the availability of resources.

### Setting

In the province of Ontario, all personal and nursing care within nursing homes is funded by the provincial government (MOHLTC), while residents are responsible for accommodation charges such as room and board. Accommodation costs are set by the MOHLTC and are standard across the province; however, rate reductions are available through a government subsidy for those with low income on a case-by-case basis. Prescription drug costs for individuals who reside in nursing homes are covered by the Ontario Drug Benefit (ODB) Program, provided they are prescribed by an Ontario physician or other authorized prescriber.

Two current province-wide initiatives exist to address the management of BPSD in nursing homes—the P.I.E.C.E.S.™ educational model and Behavioural Supports Ontario. P.I.E.C.E.S.™ training takes a person-centred approach to the understanding of, and care for, complex and at risk residents and is designed for healthcare providers who have a clinical responsibility for assessment and care planning within nursing homes (http://pieceslearning.com/). Behavioural Supports Ontario provides mobile services to care providers and family caregivers, including case management and transitional supports, dementia day programs, and respite care (http://www.behaviouralsupportsontario.ca/). The extent to which these standard supports are accessed is variable across homes.

### Recruitment

Details on overall study recruitment can be found in the original protocol [18]. Purposive sampling of the intervention group only (nursing homes receiving AD) was used for the qualitative evaluation to ensure variation in prescribing rates, size, and ownership. No participants were recruited from control homes for the qualitative evaluation. Homes were located in the same geographical area to ensure that all interviews could be conducted in-person. The study team made initial contact by email or phone with the Senior Management and/or the Director of Care explaining the study and requesting interviews with staff who had engaged with the AD intervention. As a first wave of recruitment, the home administrators were asked to identify individuals who had engaged with the academic detailers. As a second phase of recruitment, snowball sampling was used to seek providers with various perspectives on APM prescribing. All academic detailers were invited to participate in an interview.

### Data collection

Quantitative prescribing outcomes were assessed using the ODB database—a population-level administrative database linked through unique, encrypted patient identifiers at the Institute for Clinical Evaluative Science (ICES). The ODB covers all prescription medications dispensed to residents in nursing homes in Ontario. Outcomes included the mean antipsychotic daily dose and the proportion of residents dispensed an antipsychotic, acetaminophen, antidepressant, or benzodiazepine. All outcomes were calculated based on the previous 28 days of dispensed medication.

Semi-structured interviews were conducted with nursing home administrators, medical directors, nurses, social workers, personal support workers, and academic detailers to explore their opinions and experiences of an AD intervention to improve the appropriateness of APM prescribing in nursing homes. All interviews were conducted at the participant’s place of work at a date and time that was convenient for them.

The interview guide was informed by the Consolidated Framework for Implementation Research (CFIR) [21] and is available upon request. The CFIR provides a comprehensive taxonomy of defined constructs that are likely to influence implementation, allowing for the identification of key contextual features that contribute to intervention success [21]. The context of an AD intervention and the environment in which it is delivered may influence its effectiveness [22], underscoring the value of applying a framework to facilitate systematic exploration. A research associate with graduate-level training in qualitative research but with no prior relationship with study participants or study team members completed all interviews. All interviews were audio-recorded and transcribed verbatim.

### Data analysis

Dichotomous prescribing outcomes were analyzed using generalized linear mixed effects regression with binomial distribution and logit link function. Continuous prescribing outcomes (e.g. mean dose) were analyzed using linear mixed effects regression with normal distribution and identity link. Analyses were conducted according to both intention-to-treat and as-treated principles, as the latter was more sensitive to the true effects of the intervention and therefore more informative for policy makers. A more detailed overview of the quantitative analysis can be found in the protocol [18].

Interviews were analyzed using the framework method [23, 24], with CFIR constructs applied as pre-defined deductive codes. Open coding was applied when themes emerged that did not fit within the definitions of CFIR constructs. Transcripts were coded using NVivo 11 [25]. Several strategies were used to ensure fidelity and credibility of the data, such as using multiple sources of data; creating a chain of evidence that documents all elements of the study database; having key collaborators participate in the triangulation analysis and the return of findings (construct and external validity); examining points of convergence and divergence within and among the dataset (internal validity through cross comparative analyses); and having a stepped analysis process whereby there was an initial independent review of the data by three reviewers (LD, MS, LJ) who then met to reach consensus around the common themes (reliability) [26]. Once common themes were established across all interviews, the framework method was applied to explore the experience of each nursing home using a comparative case study technique [23]. During this phase, transcripts were indexed using existing codes and a matrix was created to visualize the presence of codes across cases (for the purposes of this study, each individual nursing home was considered an independent case). The characteristics of, and differences between, the cases were identified to generate typologies and map the connections between cases and codes to explore relationships and/or causality. Data were triangulated by mapping qualitative findings alongside divergent quantitative findings to explore potential explanations underlying quantitative results.

## Results

### Interim quantitative prescribing outcomes

**Fig. 3:**
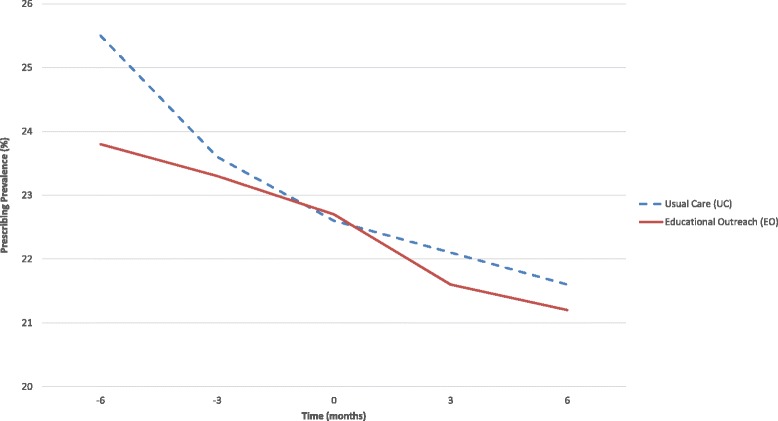
Longitudinal prevalence of antipsychotic prescribing

Prescribing outcomes are reported in Table 1. No statistically significant differences were found. The as-treated analysis revealed a decrease in overall prevalence of APM prescribing of 1.9% in the intervention group (compared to a reduction of 0.9% in usual care), with a gradual downward trend over time (refer to Fig. 3). A transient reduction in the mean daily dose of APM was observed at 3 months among those homes who participated in the intervention, compared to a slight increase in mean daily dose among those homes who did not receive the intervention.

**Table 1:** Secondary prescribing outcomes from interim analysis

Interim prescribing outcomes	Academic detailing intervention			Usual care		
	Baseline	3 months	6 months	Baseline	3 months	6 months
Per protocol	*n* = 2820	*n* = 2914	*n* = 2947	*n* = 3934	*n* = 4093	*n* = 4162
Antipsychotic daily dose in the past 28 days (mean ± SD)^a^	120 ± 155	116 ± 136	122 ± 152	127 ± 156	133 ± 160	125 ± 156
Antipsychotic [*n*(%)]	639 (22.7%)	630 (21.6%)	624 (21.2%)	890 (22.6%)	903 (22.1%)	898 (21.6%)
Acetaminophen [*n*(%)]	1237 (43.9%)	1263 (43.3%)	1244 (42.2%)	1677 (42.6%)	1749 (42.7%)	1736 (41.7%)
Antidepressant [*n*(%)]	1436 (50.9%)	1478 (50.7%)	1495 (50.7%)	2000 (50.8%)	2085 (50.9%)	2102 (50.5%)
Benzodiazepine [*n*(%)]	261 (9.3%)	275 (9.4%)	273 (9.3%)	427 (10.9%)	469 (11.5%)	414 (9.9%)
As-treated	*n* = 2342	*n* = 2421	*n* = 2453	*n* = 4412	*n* = 4586	*n* = 4656
Antipsychotic daily dose in the past 28 days (mean ± SD)^a^	118 ± 151	114 ± 132	117 ± 145	128 ± 158	132 ± 160	128 ± 159
Antipsychotic [*n*(%)]	556 (23.7%)	548 (22.6%)	535 (21.8%)	973 (22.1%)	985 (21.5%)	987 (21.2%)
Acetaminophen [*n*(%)]	992 (42.4%)	1,006 (41.6%)	988 (40.3%)	1,922 (43.6%)	2,006 (43.7%)	1,992 (42.8%)
Antidepressant [*n*(%)]	1160 (49.5%)	1200 (49.6%)	1210 (49.3%)	2276 (51.6%)	2363 (51.5%)	2387 (51.3%)
Benzodiazepine [*n*(%)]	232 (9.9%)	243 (10.0%)	238 (9.7%)	456 (10.3%)	501 (10.9%)	449 (9.6%)

^a^Among those who used continuously

### Qualitative process evaluation

A total of 22 interviews were conducted, including four academic detailers and 18 nursing home staff across five nursing homes (home characteristics can be found in Table 2). All but one of the participants were female (participant characteristics can be found in Table 3). The interviews ranged from 15 to 70 min in duration. The analysis identified key constructs from the CFIR [21] that helped to explain how and why the AD intervention did or did not work in the context of nursing homes (refer to Additional file 1 for a definition of relevant constructs). Themes corresponding to CFIR constructs have been *italicized* throughout the paper for ease of identification. In addition, themes emerged beyond the scope of the CFIR that illuminated the breadth of factors influencing APM prescribing at the resident level and those that provided considerations for scale.

**Table 2:** Characteristics of Intervention Exposure across Homes

	LTC home				
	A	B	C	D	E
Home characteristics					
Ownership	Private	Private	Public	Private	Public
No. of beds	161	132	378	140	205
Detailing visits					
Total detailing visits (*n*)	5	1	49	13	4
One-on-one visits (*n*)	5	1	19	8	2
Group visits (*n*)	0	0	30	5	2
Total duration of detailing visits (min)	290	30	1247	375	260
Total providers engaged (*n*)	5	1	111	23	8
Other visits					
Interactions^a^ (*n*)	1	3	7	8	1
Total interaction time (min)	10	25	651	100	5
Formal presentations (*n*)	2	0	5	10	3
Total presentation time (min)	125	0	170	215	100
Total duration of intervention events (min)	425	55	2068	690	365

All data presented above reflects intervention exposure at the time of the interviews. LTC homes A and E received additional detailing visits following the qualitative interviews

^a^Interactions are defined as in-person, telephone, or email exchanges that deliver content beyond the key messages

**Table 3:** Characteristics of interview participants

ID	Role	Years employed at current LTC facility	Total years of experience in LTC
AD1	Academic detailer	–	–
AD2	Academic detailer	–	–
AD3	Academic detailer	–	–
AD4	Academic detailer^a^	–	–
ID5	Medical director and attending physician	12	17
ID6	BSO RN	10	13
ID7	Home administrator	5	28
ID8	Director of care (RN)	24	24
ID9	Medical director (Physician)	4	4
ID10	Home administrator (manager of quality)	2	22
ID11	Director of care (RN)	3	16
ID12	Home administrator (RN)	8	8
ID13	Assistant director of care (RN)	3	7
ID14	BSO lead RPN	9	12
ID15	Social worker	3	3
ID16	Personal support worker	10	15
ID17	Personal support worker	6	10
ID18	Personal support worker	4	4
ID19	Director of care (RN)	14	16
ID20	RN	21	21
ID21	RPN	12	21
ID22	Personal support worker	11	35

^a^AD4 resigned as an academic detailer 2 months after the intervention launched

*BSO* Behavioural Support Outreach, *RN* registered nurse, *RPN* registered practical nurse

### Understanding the context

Academic detailers and nursing home staff described a range of system, facility, provider, and resident level factors that influence APM prescribing practices. These features aligned with constructs outlined within the CFIR themes of *Outer Setting*, *Inner Setting*, and *Characteristics of Individuals*. Participants highlighted the importance of understanding these contextual features, which interact with the intervention to produce an effect (either positive or negative). This myriad of factors may contribute to differential effects across homes, and therefore must be considered in order to understand how and why the AD intervention did or did not work.

#### Features of the outer context that influence APM prescribing

The majority of system-level factors fit within the constructs of *External Policy and Incentives* and *Peer Pressure*. The most notable system-level barrier to behaviour change was competing priorities, which included a series of mandatory initiatives and directives from governing bodies that impact direct care providers. These external expectations to ensure APM prescribing that is used for the appropriate indications were accompanied with an overarching objective to decrease overall APM prescribing rates, which often conflicted with the routine ways of managing BPSD in nursing homes, creating a barrier to behaviour change. Public reporting of variation in home-level APM rates heightened awareness about APM prescribing practices, and introduced external and peer pressures on providers within the nursing home sector. These pressures created a tension within the nursing home sector, shifting the focus from the individual residents to home-level prescribing rates.

“We were asked by the board to make this a priority, and then the deputy minister for the ministry of health literally sent out letters saying if you haven’t made this a priority, make it a priority, or work harder to make it a bigger priority. So there was a lot of this kind of political pressure.” (ID9, Medical Director)

Some families are very good and then there’s others that are leery and, you know, they see the negativity of what’s going on in [a nursing home], in the media, and that plays a huge role so we have to kind of overcome, you know, that. (ID8, Director of Care)

Participants explicitly identified the role of the MOHLTC and the media, with the latter simultaneously raising awareness of inappropriate prescribing of APM medications and escalating public concern. Public and media attention adopted a negative perspective, focusing on the adverse consequences of APM prescribing without acknowledging proper management, creating a culture of fear.

“The media had kind of expressed a lot of concern about the use of APMs in the nursing homes but then that caused a lot of fear for the doctors to even prescribe it, and then it became a point where the nursing staff could [experience] possible safety issues.” (AD3, Academic Detailer)

#### Features of the inner context that influence APM prescribing

Facility level factors aligned with the constructs of *Networks and Communication* and *Culture.* These factors depict the landscape of nursing homes as one of variability, fragmented communication processes, and time constraints. In conversations with the detailers, participants highlighted the communication and documentation processes that contribute to APM prescribing specifically needed attention.

“It’s a bit fragmented, it depends on the home. I would say the worst case scenario is when the Personal Support Workers (PSWs) don’t get the respect from the registered staff and then the registered staff say well, “the PSWs aren’t giving me the information so I can’t document properly”, and then they tell the physician we need an APM or we need a drug to sedate this person because we can’t care for this person, so and there’s a lot of miscommunication with what is really the issue with the resident.” (AD3, Academic Detailer)

The majority of participants expressed that the current state of APM use required change. Aligning with the *Tension for Change* sub-construct, this desire for change was driven by the consideration of current prescribing rates in combination with external media and MOHLTC pressures. Among homes who experienced this tension for change, participants reported the presence of engaged leaders within the home who were committed to improving quality.

“To be honest there were a lot of emphasis on indicators but I didn’t see [APMs] as an indicator until I started looking at that article and then I started looking at the research and then I started looking at evidence-based practice guidelines and I said, well, we have a serious problem. So, what we did, we started an initiative immediately without anybody saying that we had to. So, we did, we looked at the percentage of our residents, who were on antipsychotics without the diagnosis of psychosis.” (ID12, Home Administrator)

The influence of medical and administrative leaders was reported to be an important factor contributing to the organizational culture of APM prescribing. Self-monitoring of home-level prescribing rates was often used to drive change. In an attempt to reach prescribing goals, providers often pursued additional training related to APM prescribing; however, many participants described persisting gaps in practices.

“We have a funded BSO team however, I still don’t think that to be just given, because I am [involved with BSO] and when we do our rounds together and I see the nurses and how they intervene, I still find that there’s a gap with antipsychotics. I still think that they are heavily relying on it.” (ID15, Social Worker)

The reality of resource constraints and limited time compound the pressures exerted on nursing home administrators and providers. Resources available to help overcome these barriers and address APM prescribing were described as fragmented and siloed, often resulting in persisting knowledge gaps and suboptimal impact on practice.

“We are still lacking of more knowledge in regards with the non-intervention. Because I know I always send my frontline staff for P.I.E.C.E.S., you know that kind of training, but they are not practicing it. But because of this academic detailing, you know, I don’t need to send them, because we have guidelines now, and then because of that education that [the detailer has] given to my staff, it helps them a lot.” (ID11, Director of Care)

#### Characteristics of individuals that influence APM prescribing

Gaps in practice were identified at the provider level and were associated with the *Knowledge and Beliefs* construct. Gaps included a lack of knowledge around indications for antipsychotics, associated harms, and alternative approaches for managing behavioural symptoms. Prescribers often do not know or have adequate information of the BPSD being displayed by residents. In such cases, medication related-decisions are made based on information provided by the direct care providers (e.g. nurse and/or personal support worker) who also struggle with time constraints and knowledge-related barriers.

“Because with the anti-psychotic initiative, for a physician coming into a [nursing home] facility, [they] usually look at the [registered practical nurse] who’s all harassed and harried. And she’ll say ‘So and so has behaviours.’ And it’s very easy for them to say ‘Okay, you know, we’re going to just, we’re going to put them on an anti-psychotic.’ But he doesn’t have the information that he needs to make that appropriate prescription, right?” (ID19, Director of Care)

Additional challenges that impact both the prescribing and administration of APM did not align with the CFIR themes, and relate to the complexities of managing residents in nursing homes. Participants highlighted the challenges of resident acuity, managing multiple comorbid conditions, polypharmacy on admission without accompanying documentation, and high resident turnover rates. The reality of managing complex residents was described as a barrier to avoiding the prescription of high-risk medications within the nursing home sector.

### Exploring academic detailing: how and why does it work (or not) in this context?

#### Credibility and a tailored approach: intervention characteristics drive engagement

Several constructs within the *Intervention Characteristics* theme contributed to participant engagement, including *Intervention Source*, *Evidence Strength and Quality*, and *Adaptability*. Detailer credibility (*Intervention Source*) was a critical component of engaging participants in the intervention and was subject to differences in detailers, participants, and encounters. Credibility was established through a combination of demonstrated knowledge, understanding of the nursing home context, and confidence, and did not seem to be related to the detailer’s professional discipline (e.g. nursing versus pharmacy). The detailer’s “third party” perspective enhanced their credibility in the home and served to provide external validation of efforts made to improve appropriateness of prescribing. Detailers were viewed by nursing home staff as a neutral party who helped refocus the homes’ APM prescribing efforts back to a clinical lens in the wake of recent media and ministry attention.

“The academic detailer, you know, explained the reasons why it was so valuable, because it kind of helps to establish a pattern, and whether or not medication is working successfully for that person, you know, resident, and whether de-prescribing is necessary. Again, it helped to kind of reinforce and validate the reasons why. Because although we provide information, as the leadership team here at the [home] … hearing it from the third party, was like ‘oh yeah, it is really important’.” (ID10, Manager of Quality)

Participants viewed the detailer’s ability to be approachable and flexible as key characteristics underlying relationship development *(Adaptability)*. For example, staff valued when the detailer was willing to answer questions unrelated to the primary topic of APMs. Strong interpersonal skills were emphasized as an integral part of successfully engaging staff across the home. Tailoring the approach to meet the needs of each home and provider was a critical feature of the intervention that helped detailers to achieve buy-in at the home and individual level. Underlying a flexible approach was a strong evidence base (*Evidence Strength and Quality)* that contributed to participant engagement, the credibility of the intervention, and the value of the educational material.

“I am encouraging everyone [to participate] because when I look at [the intervention], everything is evidence-based. Not everything is evidence-based, it’s trial and error, so how I sell these [clinical] practices is you look at evidence-based practice because it has been tried and trialed and then put into effect.” (ID12, Home Administrator)

“When I first opened the 8 page guide I thought wow, there’s a lot of information here for somebody to present to me, but I didn’t get that back. I really got you know, high perceived value and, and a well-researched project and I think a lot of confidence in the evidence and, and what was presented.” (AD1, Academic Detailer)

Two homes reported a negative perception of the detailer when credibility was not established. One home felt that they might still benefit from the intervention under different circumstances while the other home was confident in their ability to address the topic of APM prescribing without external support. The detailer reflected the sentiments of these homes, citing a lack of contextual experience and comfort level as a reason for poor credibility.

“My lack of experience and my lack of confidence was a barrier as well. Like I, I really do think that it’s an individual intervention that really, you know, it hinges on me, the characteristics and the experience and the abilities of the detailer.” (AD4, Academic Detailer)

#### Compatibility between intervention characteristics and the inner setting drives change

Several features of the intervention and the inner setting aligned in homes where participants described perceived value. Homes that valued the intervention exhibited a *Tension for Change* and *Leadership Engagement.* The content of the AD intervention provided *Available Resources*, *Access to Knowledge and Information*, and a *Relative Advantage*. Detailer credibility (*Intervention Source*) emerged as a critical feature of the intervention in order to successfully apply the characteristics of the intervention to the inner setting.

“We were lacking more knowledge in regards [to non-pharmacological management]. Because I know I always send my frontline staff for P.I.E.C.E.S., you know that kind of training, but they are not practicing it. But because of this academic detailing, you know, I don’t need to send them, because we have guidelines now, and then because of that education that [the detailer] has given to my staff, it helps them a lot.” (ID11, Director of Care)

When the relationship with the detailer was strong (*Intervention Source*), homes reported that the intervention had a high perceived value, exceeding their expectations. In contrast, the two homes who reported a negative perception of the detailer noted a lack of perceived value in the overall AD intervention. Nursing home staff valued education around non-pharmacological approaches to managing BPSD, alternatives to APM, and detailing within the context of each respective nursing home to allow participants to be effective change managers (*Access to Knowledge and Information* and *Available Resources*). It is important to note that changes in practice were only reported for those professional groups who directly participated in AD sessions. Homes reported more comprehensive changes when front-line staff were engaged in addition to administrators, physicians, and pharmacists.

“I think having that dialogue in front of the PSW has been really valuable, and I did that because, whereas previously a lot of PSWs felt very uncomfortable with this topic, they are now engaged, they are interested, they were committed, and families trust PSWs and nurses above physicians (laughs) and so having a unified team was really valuable.” (ID9, Medical Director)

Several participants noted that, by engaging staff from a variety of professional backgrounds and roles, the AD intervention unified the home and strengthened the home’s quality improvement efforts. The ability of the AD intervention to include all provider groups across the home was viewed as a unique feature when compared to other available resources (*Relative Advantage*). Participants also reported natural spread, which included presentations to board members, family councils, requested attendance at regional meetings, and recommending the intervention to other nursing homes not involved in the intervention. Detailers felt the success of the intervention hinged on their ability to identify who or what drives the prescribing patterns and utilized their topic specific knowledge to address perceived gaps.

“They will say “well, if this nurse is on I’ve got the best information so I know I need to go down and detail her” right? So, it kind of snowballs into an ‘Ah, ha!’. So if she is the girl you rely on let’s go speak to her and find out, what her attitudes are towards appropriate prescribing and or de-prescribing.” (AD2, Academic Detailer)

Homes consistently reported that *Leadership Engagement* and *Tension for Change* were underlying drivers of the change within the context of the AD intervention. Engaged leaders, often the medical directors, were viewed as internal champions for improving the appropriateness of APM prescribing across the home. Homes with high baseline prescribing rates identified a strong tension for change that motivated them to engage with the AD intervention and apply new knowledge to practice. In addition, there was a sense of compatibility between established priorities and work processes within the nursing homes and the structure of the intervention, further contributing to a climate for change.

“We have been fortunate because [our Medical Director] is always one to compress meds and not, you know. Our residents come in with massive amounts of medication, and some of them don’t even know why they are on certain meds and so we do certainly look at decompressing when we can. So, we’re fortunate to have someone that’s, you know, got that mindset and is dedicated.” (ID8, Director of Care)

### Moving forward and sustaining change

#### Perceived impact on outcomes proximal to prescribing

Both detailers and nursing home staff believed that the intervention’s impact on APM prescribing rates will not likely demonstrate an impact on prescribing rates within the 6-month intervention period due to the cascade of factors that influence prescribing. Participants reported that the intervention had an impact on a variety of outcomes that are precursors to APM prescribing, including those at the level of the nursing home and the individual provider. At the home level, participants reported several areas of impact, including improved documentation processes, the introduction of a common language, increased use of non-pharmacological approaches, and more collaborative practice within the home.

“Before the program we were not that cautious of writing the reason on the orders of medication […] We were reviewing every three months and we were thinking “Okay, it’s working, it’s not working”, well, we didn’t have the reasons there. So, since this program came to us it gave us that idea, then we are starting now in every three month review, when we do it with the doctors, make sure that we write the reason behind, and we know exactly why the medication is used.” (ID5, Medical Director & Attending Physician)

“I think having that dialogue in front of the PSW has been really valuable, and I did that because, whereas previously a lot of PSWs felt very uncomfortable with this topic, they are now engaged, they are interested, they were committed, and families trust PSWs and nurses above physicians (laughs) and so having a unified team was really valuable.” (ID9, Medical Director)

The intervention also helped to strengthen pre-existing initiatives (*Compatibility*), which motivated the homes to continue their efforts in addressing inappropriate prescribing. At the provider level, participants noted increased awareness around indications for APM use, improved motivation, increased knowledge, and a more comprehensive understanding of the topic. With a comprehensive understanding, nursing home staff were able to discuss the topic of APM use with family members, which positively impacted both the family and residents’ experience.

“There was a gentlemen [who] was on a lot of antipsychotics and the family had fear of taking him off it because they were educated as well that they need it, it’s keeping them calm, this person was on it for a while. I asked the family plus the [physician] if we can just try, its trial and error if we see that he is getting worse … we put them back. We’ve taken the resident off slowly and before the resident was not able to talk, drooling, comatose, sleeping and I didn’t know he could have talked, and when we took him off the medication oh my gosh, it brought tears to my eyes. He would go, “I want coffee” … where before he would just sit there with a blank stare.” (ID14, Registered Practical Nurse)

#### Recommendations for scale

Participants offered several recommendations for consideration if the AD intervention was to be scaled up across nursing homes in Ontario. Academic detailers explained the difficulty they experienced when attempting to engage homes with a single administrative contact as the gatekeeper, reporting it was much easier to engage homes when they had direct access to staff. Detailers also saw a need to plan for variation in the amount of resources provided to each home depending on its needs on both individual and home levels.

“One of the things that in future I would like to kind of work through differently … we rely on the long term care home to say, “oh and here’s the name of your pharmacist, here’s the name of your physician” and sometimes having that long term care home as the gatekeeper of who you can talk to can delay your ability to get in and get all of the detailing done close together.” (AD2, Academic Detailer)

Nursing home staff expressed the need to fill perceived gaps around the practical application of the evidence provided by the detailers, which may suggest that future AD interventions would have a greater impact if they provided explicit guidance on what to do for specific cases. Participants from smaller homes expressed the value in establishing communities of practice for those homes who do not currently belong to a larger network in order to optimize the spread of best practices. Finally, for complex topics like APMs, participants perceived value in engaging family members in AD visits to help improve the resident experience and deliver client-centred care.

“I just think we should change with the generation. Instead of staying back, we should move forward. We should educate everybody, not only the staff. Educate the family. Let them know about these medications. Let them know.” (ID18, PSW)

## Discussion

This study used a mixed-methods approach to (1) quantitatively evaluate the impact of an AD intervention on (interim) prescribing outcomes and (2) qualitatively explore how healthcare professionals and administrators in nursing homes experienced the AD intervention to interpret quantitative findings. The key findings provide insight into contextual factors that influenced engagement and perceived impact, intervention factors that promote engagement, and the mechanisms by which the intervention seems to impact prescribing behaviour. Responding to the call to link CFIR constructs to intervention outcomes [27], our findings also highlight the set of constructs that facilitated perceived success of the AD intervention (Additional file 2). Taken together, the results suggest that 6 months may not be enough time to observe the potential effects of the intervention, but it may be enough time to appreciate how the intervention may work and to identify areas of opportunity. Complex interventions targeting complex problems are inherently dynamic and influenced by the context in which they are delivered [28]. A failure to appreciate contextual differences and understand the breadth of factors contributing to a given problem often results in suboptimal impact [28], underscoring the need for a flexibly intervention. Qualitative results demonstrate that the *Adaptability* and *Compatibility* of the AD intervention were critical to ensure its ability to target the complexities surrounding prescribing behaviour and the context of nursing homes. Intervention engagement was driven by detailer credibility, which was established through contextual knowledge, perceived expertise, and confidence, combined with a tailored approach. Participants reported a primary impact on *Networks and Communication*, including documentation and the introduction of a common language across disciplines with regard to management of residents with BPSD. The results support the use of AD as a strategy to address complex prescribing behaviours where a myriad of factors influence decision-making. Most notably, they highlight the critical features of detailer credibility, C*ompatibility*, and *Tension for Change*, which will help to inform AD interventions in similar contexts.

Prescribing decisions in nursing homes are influenced by a variety of factors across multiple levels, most notably the reality that these decisions depend on several staff members. As a result, the quality of prescribing decisions often reflects the quality of communication, underscoring the need to address poor communication as a proximal driver of prescribing practices [29, 30]. It remains unclear whether these reported changes in the attitudes and clinical decision-making of direct care providers may influence prescribing outcomes; interim analyses detected no significant differences, but primary analyses may be more sensitive to change. Previous studies have reported similar findings, with quantitative data revealing variable changes of questionable significance while qualitative findings suggest a shift in clinical thinking [28]. It is important to note that, although no between-group differences were observed, the desired outcome of a reduction in APM prescribing was observed as secular trend across both groups. Recipients of the AD intervention perceived a value-add compared to usual care (no external support), citing that the intervention successfully addressed communication processes at both the provider and home level. These qualitative insights illustrate the intervention’s compatibility with the underlying problem and potential promise as a quality improvement strategy; however, the amount of attention paid to APM in the nursing home sector makes it difficult to tease out the relative effect of AD. The range of contributing factors suggest value in engaging at both the home and individual level, as both process barriers and individual barriers contribute to inappropriate prescribing [29]. The ability of AD to address barriers across multiple levels aligns with best-practice evidence that interventions should be designed to target multiple levels of influence [31]. This further emphasizes the presence of *Relative Advantage* compared to other available resources in the nursing home sector, which was a feature highlighted previously by intervention recipients that has been shown to strongly distinguish between cases of high and low degrees of implementation success [32].

Several best-practice features of the AD intervention promoted engagement, including evidence-based content, communication processes, and targeting a range of care providers [33]. Clinician education and recommendations about practice change were included; however, participants identified a desire for further decision support whereby clinicians can discuss challenging cases and apply evidence directly to practice. Physicians are often able to identify prescribing problems but experience a sense of helplessness arising from a lack of best-practice evidence and “ready-made” solutions [34, 35]. Although the AD intervention incorporated the use of simulated cases in an effort to apply evidence to practice, there remains a need to address this gap by ensuring that support is provided to apply evidence to real-world case examples [33]. Providing decision support and feedback on clinical performance can act as complementary components of an AD intervention [33], highlighting an opportunity to optimize impact using available performance data (e.g. pharmacy prescribing records). Participant experiences echo previous work which found that academic detailer characteristics were important; specifically that they were independent from the pharmaceutical industry and health authorities and that they understood the complexities that surround prescribing practices [36].

The support provided by academic detailers translated the challenge of APM prescribing from a political issue to a clinical issue by shifting the focus from overall prescribing rates back to what is best for each individual resident. Establishing credibility and developing a strong relationship appeared to be necessary precursors to influencing prescribing behaviour. This may be explained by understanding that clinicians rarely access research findings and clinical guidelines to answer specific questions in a linear fashion, relying instead on “mindlines”—internal tacit guidelines informed by a variety of sources, including interactions with influential colleagues [37]. The concept of clinical mindlines posits that knowledge is created in social processes, through discourse, which includes underlying elements of conscious and unconscious sense-making [38]. As a result, solution-focused interventions that emphasize relationship building and collaborative learning may be a promising approach to foster the development of evidence-informed mindlines, offering a potential explanation underlying the compatibility of AD as an intervention to influence prescribing behaviours.

APM prescribing sits within a larger system of management of BPSD, which is influenced by a myriad of factors. Relationship building is not often a feature of initiatives in nursing homes, but was perhaps the most critical element of success. Providers often identify external factors (e.g. competing priorities and resource constraints) as the primary barriers to reducing APM use, including competing demands and resources, suggesting that the impact of provider-level factors may be under-estimated [39]. The AD intervention improved knowledge and communication at the provider level, suggesting that it may play in a role in addressing unidentified gaps by offering an opportunity for providers to self-reflect in a safe environment. This underscores the importance of taking a more diagnostic approach when addressing system-level problems to support individual change and ensure compatability on all levels [40].

### Limitations

Given the voluntary nature of participation in qualitative interviews, the results may be influenced by volunteer bias. To mitigate this, purposive sampling was used to capture the perspectives of homes that had minimal engagement with the AD intervention. Interviews were conducted 5 months after the intervention launched. Two homes (A and E) received additional AD visits following completion of the interviews; however, this additional exposure was minimal and unlikely to substantially change perceptions of the intervention. When AD interventions are sustained, participants have the opportunity to develop a relationship with the detailer. This may result in different findings and should be explored in future evaluations of AD interventions. The study may be limited by the inclusion of only five nursing homes in a confined geographical area. The inclusion of a small number of homes was necessary to achieve a depth of understanding with respect to the nursing home context, the features of the intervention, and the interactions between the two. The broad sample of providers also increases the likelihood that these findings are a true representation of the contextual factors that contribute to APM prescribing in nursing homes and the potential impact of the intervention across all members of the team. Finally, we were unable to recruit attending physicians (who did not occupy the role of medical director) or pharmacists, and are therefore unable to comment on the effectiveness of the AD intervention for these providers.

Interpretation of the quantitative results is limited as we do not have data quantifying whether and to what extent participating homes accessed additional supports (e.g. P.I.E.C.E.S training). However, as these supports are available to all homes in the nursing home sector and participants in this study expressed persisting knowledge and practice gaps despite participating in P.I.E.C.E.S training, it is unlikely that these external initiatives were responsible for the qualitative and quantitative outcomes reported in this paper. Pre-existing secular trends further complicate interpretation, as the intervention was delivered against the backdrop of a gradual, province-wide decrease in APM prescribing rates, which dropped from 33 to 23% over the last 5 years [41]. The interim outcome analysis is limited by the nature of the secondary outcomes and ODB data. Given that the study sample size was calculated according to the primary outcome (for which data is not yet available), it is possible that power for these dichotomous secondary outcomes was inadequate, resulting in an increased likelihood of type 2 error. Furthermore, the ODB database reports medications that were dispensed by the pharmacy and does not necessarily reflect whether the medication was administered to the resident (i.e. in cases where there is a standing order for APM “daily, as needed”, the ODB data would reflect daily use regardless of how often the resident actually took the medication). The dichotomous nature of the prescribing outcomes is less sensitive to changes in appropriate prescribing that is the focus of the intervention (i.e. a reduction in dose), which may underestimate impact on prescribing practices. It is important to note that the evaluation of secondary outcomes was included to allow for a timely interpretation of the process evaluation, as the availability of additional administrative databases will delay the primary analysis [18]. This was planned to support the timelines required by funders and partners and supported by the evaluation team since decisions based on suboptimal information was preferred to other options.

## Conclusions

Qualitative process evaluations may be useful during early stages of evaluation to understand how the intervention is working and how it might work better. Multiple healthcare providers are involved in management of residents in nursing homes, creating a complex list of interacting factors that affect appropriate prescribing. The flexibility of the AD intervention and the detailer’s clinical and contextual knowledge were critical to target the complexities surrounding prescribing behaviours. Improved communication processes and a common language across the team were reported as proximal outcomes which may have a downstream effect on APM prescribing rates.

## Additional files

Additional file 1: Intervention description. (DOCX 14 kb)

Additional file 2: CFIR key constructs and definitions. (DOCX 14 kb)

## Abbreviations

AD: Academic detailing
APM: Antipsychotic medication
BPSD: Behavioural and psychological symptoms of dementia
BSO: Behavioural Support Outreach
CFIR: Consolidated Framework for Implementation Research
ICES: Institute for Clinical Evaluative Sciences
MOHLTC: Ontario Ministry of Health and Long-Term Care
ODB: Ontario Drug Benefits
OMA: Ontario Medical Association
RN: Registered nurse
RPN: Registered practical nurse
