# Supplementary material for: Improving the appropriateness of antipsychotic prescribing in nursing homes: a mixed-methods process evaluation of an academic detailing intervention
Source: Implement Sci. 2017 May 26;12:71. doi: 10.1186/s13012-017-0602-z (PMC5446684; doi:10.1186/s13012-017-0602-z)
Supplement: Supplementary file 1 — Intervention description. (DOCX 14 kb) [file 13012_2017_602_MOESM1_ESM.docx]

**Appendix – Overview of Intervention Design**

The Centre for Effective Practice was responsible for developing and delivering the intervention (an academic detailing service), which offered nursing home care providers and staff service-oriented, one-on-one visits focused on delivering objective, balanced, evidence-informed information on best prescribing practices to optimize clinical care for residents living with BPSD. Information was always tailored to each provider’s expressed needs and delivered at a time and location that was convenient for them, often in person at their nursing home. During each visit, academic detailers also equipped providers and staff with evidence-based discussion guides to support clinical decision-making and material to share with residents, families and caregivers (https://effectivepractice.org/resources/academic-detailing-service/).  The academic detailers were health care providers (primarily pharmacists), who received intensive training on the academic detailing delivery model from the Australian Drug and Therapeutics Information Service, in addition to in-person training at Alosa Health in Boston, MA. In addition, the detailers participated in topic-specific training, involving academic detailing experts and nursing home physicians, to ensure in-depth knowledge of:

- The primary and secondary research for each clinical topic;
- Existing tools and initiatives related to each clinical topic; and
- The care context including common gaps and enablers for providers and staff.

Each provider was offered a visit per topic, and second visits were offered based on identified need. Key messages were developed following a literature search of existing recommendations, clinical evidence, and implementation evidence, as well as an environmental scan to identify programs and materials related to appropriate antipsychotic prescribing and managing behavioural and psychological symptoms of dementia (BPSD). From these searches, the Centre for Effective Practice extracted the key messages, building out the features, benefits, barriers and enablers for each, prior to review and revision by a clinical working group.

An eight-page discussion guide provided a synthesis of available evidence, and is publicly available through the Centre for Effective Practice website (https://effectivepractice.org/resources/academic-detailing-service/). The discussion guide was distributed actively directly to intervention homes by the detailers, but was publicly available to all nursing homes in Ontario. The primary target audience was defined as individuals with the most direct role in the prescribing of antipsychotic medications for residents and/or the implementation of home-wide interventions. This included physicians, medical directors, executive directors, directors of care, pharmacists, nurse practitioners and nurse managers. The secondary audience was defined as individuals with a direct role in the care of residents with BPSD and an indirect role in the prescribing of antipsychotic medications for these residents through documenting behaviours, including secondary target audience members included administrators, nurses, social workers, personal support workers, and other frontline providers.
