# Supplementary material for: Improving the appropriateness of antipsychotic prescribing in nursing homes: a mixed-methods process evaluation of an academic detailing intervention
Source: Implement Sci. 2017 May 26;12:71. doi: 10.1186/s13012-017-0602-z (PMC5446684; doi:10.1186/s13012-017-0602-z)
Supplement: Supplementary file 2 — CFIR key constructs and definitions. (DOCX 14 kb) [file 13012_2017_602_MOESM2_ESM.docx]

**Appendix A: Consolidated Framework for Implementation Research (CFIR) Themes & Constructs**

*Taken from: Damschroder LJ, Aron DC, Keith RE, Kirsh SR, Alexander JA, Lowery JC. Fostering implementation of health services research findings into practice: a consolidated framework for advancing implementation science. Implement Sci. 2009;4:50.*

**THEME 1: INTERVENTION CHARACTERISTICS**

**Intervention Source:** Perception of key stakeholders about whether the intervention is externally or internally developed.

**Evidence Strength & Quality:** Stakeholders’ perceptions of the quality and validity of evidence supporting the belief that the intervention will have desired outcomes.

**Relative Advantage:** Stakeholders’ perception of the advantage of implementing the intervention versus an alternative solution.

**Adaptability:** The degree to which an intervention can be adapted, tailored, refined, or reinvented to meet local needs.

**THEME 2: OUTER SETTING**

**Peer Pressure:** Mimetic or competitive pressure to implement an intervention; typically because most or other key peer or competing organizations have already implemented or in a bid for a competitive edge.

**External Policy & Incentives:** A broad construct that includes external strategies to spread interventions including policy and regulations (governmental or other central entity), external mandates, recommendations and guidelines, pay-for-performance, collaboratives, and public or benchmark reporting.

**THEME 3: INNER SETTING**

**Networks & Communications:** The nature and quality of webs of social networks and the nature and quality of formal and informal communications within an organization.

**Implementation Climate:** The absorptive capacity for change, shared receptivity of involved individuals to an intervention and the extent to which use of that intervention will be rewarded, supported, and expected within their organization.

***Sub-constructs***

***Tension for Change:*** The degree to which stakeholders perceive the current situation as intolerable or needing change.

***Compatibility:*** The degree of tangible fit between meaning and values attached to the intervention by involved individuals, how those align with individuals’ own norms, values, and perceived risks and needs, and how the intervention fits with existing workflows and systems.

**Readiness for Implementation:** Tangible and immediate indicators of organizational commitment to its decision to implement an intervention.

***Sub-constructs***

***Leadership Engagement:*** Commitment, involvement, and accountability of leaders and managers with the implementation.

***Available Resources:*** The level of resources dedicated for implementation and on-going operations including money, training, education, physical space, and time.

***Access to Knowledge and Information:*** Ease of access to digestible information and knowledge about the intervention and how to incorporate it into work tasks.

**THEME 4: CHARACTERISTICS OF INDIVIDUALS**

**Knowledge & Beliefs about the Intervention:** Individuals’ attitudes toward and value placed on the intervention as well as familiarity with facts, truths, and principles related to the intervention.
